# Silver and Its Salts as Surgical Antiseptics

**Published:** 1897-02

**Authors:** B. Credé


### Abstracts and Translations.

### SILVER AND ITS SALTS AS SURGICAL ANTISEPTICS.[1]

BY DR. B. CREDÉ.[2]

Dr. B. Credé, in an address delivered before the National Surgical Society of Germany at its Twenty-fifth Annual Convention, held at Berlin on May 28, 1896, discusses silver and its salts—more especially citrate and lactate, which are termed in Germany itrol and actol respectively—as the surgical antiseptics of the future. We present an abstract of his address:

Dr. Credé begins by remarking on the impossibility of treating the entire subject with any degree of exhaustiveness within the necessary limits of his paper, and referring those who desire more detailed information concerning it to his treatise on "Silver and Silver Salts as Antiseptics," published by F. G. W. Vogel, of Leipsic. The observations of his father, made years ago, on the value of the nitrate of silver in the treatment of the inflammatory affections of the eyelids in infants, had led him to make a general inquiry into the therapeutic application and usefulness of silver and its salts. He found, however, that so far as wounds were concerned, he had no success with the nitric acid salt of the metal, on account of its chemical instability and its corrosive action on the mucous membranes. Nor did his trials with the argentic albuminoids, more especially with argonine, give any better results. While visiting the Johns Hopkins Hospital, in Baltimore, however, he was struck with the good results obtained by Dr. Halsted by the use of silver foil as an antiseptic covering for small or closed wounds.

In a subsequent series of experiments made with his assistant, Dr. Beier, Credé proved that metallic silver, when placed upon aseptic sterile wounds, is non-irritating and remains unchanged, forming a thoroughly aseptic dressing. On the other hand, it appeared that when a wound or any part of it was infected, the

[1] Abstract of an address delivered before the National Surgical Society of Germany at its Twenty-fifth Annual Convention, held at Berlin on May 28, 1896.

[2] Royal Councillor and Chief Surgeon to the Carola Hospital of Dresden.

products of bacterial vitality oxidized the surface of the silver, and, entering into combinations with the argentic oxide, formed argentic albuminates which had powerful antiseptic properties. Credé and Beier succeeded in determining that it was the organic acids, and especially lactic acid, formed in the microbic secretions, that united with the silver oxide, and they proved that an infected wound, when dressed with metallic silver, generated an antiseptic "lactate of silver." At Credé's suggestion the Chemische Fabrik von Heyden, at Radebeul, near Dresden, prepared a pure and absolutely stable lactate of silver.

Lactate of silver is a white, odorless, almost tasteless powder, permanent if kept in a brown glass vial, and soluble in fifteen parts of water and albuminous fluids. It has no irritating or corrosive action upon wounds, though in sensitive cases it caused more or less burning. In 1 : 1000 watery solution it destroyed streptococci, staphylococci, and the anthrax bacillus within five minutes. In blood-serum it retarded the development of bacterial germs in a dilution of 1 : 80,000; whilst corrosive sublimate does so only in a strength of 1 : 20,000. The destructive action of the silver salt on bacterial life is, therefore, four times as great as that of corrosive sublimate, as has been already noticed by Koch and Behring. And not only is corrosive sublimate far more poisonous than the silver salts, but it has the great disadvantage of forming insoluble compounds with albuminous substances when applied in concentrated solution, whereby it destroys the cellular tissue and a further penetrating antiseptic action is prevented. Whilst the silver salts prevent the growth of the bacteria, they do not destroy the cellular tissue; they remain in solution, and the solution permeates the tissues layer by layer. The lactate of silver being absolutely non-poisonous, and both it and its albuminates remaining soluble, may be used hypodermically in the infectious diseases. Credé treated two hopeless cases of anthrax and five grave cases of erysipelas by the subcutaneous injection of a solution of lactate of silver. He used .5 : 20 (¾ grain to 5 drachms) of water for the anthrax and .3 to 1 : 100 to 200 (5 to 15 grains to 3⅓ to 6⅔ ounces) of water for the erysipelas cases. All cases recovered. Encouraged by his success, he proposes to use this treatment in cases of septicæmia, pyæmia, puerperal fever, and diphtheria. The injection is somewhat painful and should be done under cocaine or general anæsthesia.

In veterinary practice a solution five times as strong may be employed.

Eight other organic silver salts were prepared by the Chemische Fabrik von Heyden for Dr. Credé in the attempt to obviate the irritant effect that the lactate of silver shows when employed in powder form, on account of its ready solubility. It was thought that on account of their close chemical relations to that salt they would have similar antiseptic properties, whilst being less readily soluble there would be less possibility of any toxic effect when freely used for long periods. Clinical and bacteriological experimentation both showed that the citrate of silver was the most efficient form of the metal.

Citrate of silver is a light, dusty, and stable powder, without odor and almost devoid of taste. It has the same antiseptic power as the lactate, but it requires three thousand eight hundred parts of water for solution. A watery solution of 1 : 4000 destroys all bacteria within ten minutes. It occasions no unpleasant or painful sensation at all in any wound, and its scanty solubility causes it to last longer and enables it to be employed in smaller quantities. It is, therefore, cheaper to use than iodoform, though its price is more than twice as great.

Credé has now used the citrate of silver for more than seven months, and has treated with it over four hundred surgical cases and over one thousand patients in private and clinical practice. He has never observed any abnormal or detrimental action; on the contrary, there has been a normal and rapid process of healing never before experienced with the former methods of asepsis and antisepsis.

Credé then proceeds to detail his method of treating wounds. Whilst recognizing the desirability and value of an aseptic course of treatment, he rightly claims that the antiseptic one cannot be dispensed with, since many wounds come to the surgeon already infected, others are so situated that aseptic treatment is impossible, and a perfect aseptic treatment is only possible in a well-appointed hospital, not being practical in private practice, and still less on the battle-field. He has frequently observed in parallel cases that the process of healing was much more rapid with his antiseptic method than with the ordinary aseptic one, besides being less expensive on account of the relatively small amounts of material used. Chemical disinfectants are, it is true, going more and more out of use, probably because no universally applicable antiseptic is yet known. There is no one that is entirely devoid of irritating action, that is non-poisonous, that does not affect the cellular tissue unfavorably, that is odorless, that can be used as a powder and at the same time never fails to destroy the germs. He does not maintain that citrate of silver is such an ideal antiseptic; but he does claim that we have as yet no better and no more perfect one at our command.

Credé's method of wound treatment stands about half-way between the aseptic and the antiseptic processes. After operation the wound and its surroundings are carefully and repeatedly rinsed with water. When the wound is closed it is first covered with silver gauze or with the "gray silver dressing," so called to distinguish it from the "white silver dressing employed in minor wounds and transplantations. It is a mull impregnated with metallic silver in a most minutely-powdered form; it is perfectly non-irritating, and forms undoubtedly a superior aseptic dressing. Its antiseptic action is initiated after bacterial infection of the wound and development of the lactic and other organic acids, forming the powerful antiseptic, the lactate of silver. It forms a constant and prompt counteractant to any secondary infection.

Wounds that are not to be closed at once are first dusted with the citrate of silver and then covered with the silver gauze. Clean wounds and complicated fractures are thoroughly cleansed with soap and water and liberally sprinkled with the dry citrate of silver, without disturbing the process of healing by a minuter examination; the cavity is then filled with the silver gauze and the whole covered with the common sterilized gauze. Even if the infection is already deep, a certain amount of disinfection can be accomplished in this way, for the citrate of silver remains dissolved in the serum, penetrates the cellular tissues, and reaches the point of infection. A still farther-reaching disinfection may be gotten by using the more soluble lactate of silver, as in the cases of anthrax and erysipelas above mentioned.

For rinsing cavities, such as the bladder, etc., Credé uses the citrate of silver 1 : 4000 to 10,000 of water; but for abscesses where there is much pus and where an energetic action is required, he employs the more soluble lactate of silver, 1 part in 500 to 2000 parts of water.

It is a special gratification to him to be no longer obliged to employ iodoform, which is, apart from injections in cases of tuberculosis, but too often an unreliable and insufficient antiseptic.

Pure silver materials only must be employed whenever anything is to be left in cavities or wounds for any length of time. Where silver wire cannot be employed, silk, catgut, and caoutchouc ligatures covered with metallic silver is used, and drainage-tubes are similarly coated. They thus remain sterile, and any subsequent infection entering through the drainage-tube or from the surrounding tissues is destroyed as long as the silver lasts. For the details of the preparation of these materials, as well as for the application of the citrate of silver in gynæcological, ophthalmic, aural, laryngeal, and genito-urinary practice, Credé refers to the larger treatise mentioned above.

In conclusion, Dr. Credé exhibited the following:

1. White silver dressing.
2. Gray silver dressing.
3. Silk, catgut, and drain coated with silver.
4. Petri's disks, containing—
   - *a.* Silver upon agar-agar infected in sterile strata with staphylococci.
   - *b.* Gold, silver, and copper upon a similarly infected agar-agar plate. Gold has remained intact, silver is partly, and copper is entirely dissolved.
   - *c.* Staphylococcus culture upon agar-agar, upon which has been placed a trace of lactate of silver, forming large sterile strata.
   - *d.* Disk similarly prepared and charged with a trace of citrate of silver, also forming large sterile strata.
   - *e.* Gauze, threads, and drains upon infected agar also forming large sterile strata.
5. Two drawings of silver with sterile surroundings infected with streptococci.
6. Citrate of silver and lactate of silver in dry powder.

